# Impact of combining magnetic resonance images and radiographs on radiographic grading of sacroiliitis and classification in axial spondyloarthritis: a reliability study

**DOI:** 10.1007/s00296-025-05898-0

**Published:** 2025-05-26

**Authors:** Kemal Erol, Kevser Orhan, Işıl Yurdaışık, Salih Özgöçmen

**Affiliations:** 1 Division of Rheumatology, Faculty of Medicine, Selçuk University, Konya, Turkey; 2 Division of Rheumatology, Ankara City Hospital, Ankara, Turkey; 3 Department of Radiology, Faculty of Medicine, İstinye University, Gaziosmanpaşa Hospital, İstanbul, Turkey; 4 Department of Rheumatology, Faculty of Medicine, İstinye University, Gaziosmanpaşa Hospital, İstanbul, Turkey

**Keywords:** Spondyloarthritis, Sacroiliitis, Radiograph, Magnetic resonance, Reliability, Multimodal imaging

## Abstract

To investigate the reliability of sacroiliac radiographic grading based on the modified New York criteria, both through grading pelvic radiographs alone and in combination with sacroiliac magnetic resonance imaging and the influence of alone and multimodal interpretation on the categorization of radiographic and non-radiographic axial spondyloarthritis (r-axSpA and nr-axSpA). Radiographs of the SI joints from patients with axSpA were graded by two rheumatologists independently in two sessions (radiographs only and MRI + radiographs) and followed by consensus sessions with the participation of a radiologist. Overall, anteroposterior pelvic radiographs and T1 weighted MR scans of the sacroiliac joints from 138 patients with axSpA were eligible. Intra- and inter-reader reliability was fair to moderate for both techniques regardless of whether they were examined alone or in conjunction with MRI, however confidence levels of readers were relatively higher in the MRI + radiographs combined technique compared to radiographs only. Regarding the classification, both readers’ intra and inter reader agreements were moderate to substantial. The kappa values and agreement were notably higher for the combined reading technique. In the consensus sessions, 10.2% of patients were recategorized. Merging the information from MRI scans with pelvic radiographs yielded no notable enhancement in the accuracy of radiographic grading of SI joint. Categorizing axSpA as either radiographic or non-radiographic based on pelvic radiography falls short in reliability even after combining with MR images and may likely call for a more reliable definition, axSpA with or without structural changes, relying on more advanced imaging techniques than radiographs.

Axial spondyloarthritis (axSpA) is a chronic inflammatory condition that primarily affects the spine and sacroiliac joints. It encompasses a spectrum of patients with radiographic sacroiliitis or radiographic axSpA (r-axSpA) as well as those without radiographic sacroiliitis, non-radiographic axSpA (nr-axSpA). Radiographic evaluation and magnetic resonance imaging play a crucial role in the assessment and management of this condition [1-3]. 

Conventional radiography has been a mainstay in the diagnosis and monitoring of axSpA. This imaging modality can detect characteristic radiographic changes, mainly the structural changes, in the sacroiliac joints, such as joint space narrowing, subchondral sclerosis, and erosions, which are hallmarks of sacroiliitis and can be used to diagnose the condition [1,4, 5]. Axial Spondyloarthritis includes all kinds of patients with both radiographic and non-radiographic disease, with radiographic proof of sacroiliitis being the only distinguishing feature. Identifying radiographic sacroiliitis is deemed challenging due to the complex pelvic anatomy, the oblique orientation of the SI joints, and obstructed visualization caused by bowel gas [6]. Additionally, the irregular articular surface makes it difficult to capture clear images of the SI joints on traditional radiographs, leading to misinterpretations [6, 7].

Additionally, radiography can also be used to assess the progression of the disease over time, inflammation on MRI may be a predictor for further radiographic progression in patients with axSpA [8–10]. However, radiographic changes can be subtle in the early stages of the disease and may not be readily apparent on plain films. Also, numerous studies have consistently indicated a lack of consensus among skilled readers in the radiographic grading of sacroiliac joints (SIJ), with kappa values averaging approximately 0.5 [11–13] and even could not been substantially improved with training according to an earlier study [14]. However, recent research has demonstrated a considerable improvement in agreement on scoring radiographs using the mNY criteria when an online real-time interactive calibration (RETIC) module is used in conjunction with the slideshow and video [15]. Furthermore, artificial intelligence (AI), in particular deep learning, has been progressively utilized in axSpA to enhance diagnostic evaluation by using various imaging techniques such as radiography, CT, and MRI [16, 17]. Bressem et al. [18] demonstrated a strong agreement between the reference agreement and the neural network evaluation of sacroiliitis on X-ray, with kappa values of 0.79 and 0.72 for the test and validation cohorts, respectively. The idea of anatomy-centered deep learning, which seeks to improve the signal-to-noise ratio by extracting the sacroiliac joint, eliminating additional confusing elements on the radiograph, and concentrating exclusively on pertinent anatomy, was recently applied by Dorfner et al. [19]. They demonstrated that deep neural networks can be enhanced in their diagnostic accuracy and generalizability for detecting radiographic sacroiliitis by including anatomy-centered deep learning. Additionally, using the anatomy-centric model, it was possible to predict the progression from non-radiographic to radiographic sacroiliitis with greater accuracy (odds ratio = 2.2) than using the conventional model (OR = 1.2) [19].

Magnetic resonance imaging has emerged as a valuable tool in the evaluation of axial spondyloarthritis. Over the last few years, MRI has gained widespread acceptance for identifying active inflammatory lesions by utilizing T2-weighted sequences with fat suppression, such as the short-tau inversion recovery (STIR) sequence [20, 21]. In recent years, MRI has demonstrated its superiority in detecting erosions and illustrating periarticular and intra-articular fatty metaplasia, as well as active inflammation of bone marrow and soft tissues [22, 23]. However, there are some challenging issues and limitations in the sacroiliac MRI. Structural lesions in axSpA can be difficult to interpret and may show considerable variation between readers [24, 25], inflammatory lesions may arise in healthy individuals or in patients with non-SpA low back pain [26, 27], and anatomical variations in the sacroiliac joints may complicate interpretation [28].

In everyday routine, radiologists and rheumatologists in the local settings review MRIs and X-rays of the SI-joints (multimodal interpretation), often with information of the patient’s clinical signs and symptoms. Recent data supported the idea that the diagnostic confidence and accuracy of radiologists improved when clinical information is accessible during radiologic interpretation [29]. However, in research settings and medical trials, at least one or more trained central readers (unaware of the patient’s clinical details) evaluates the images. Since the diagnosis of axSpA relies heavily on the existence of sacroiliitis, a patient’s classification could potentially be changed if another reader interprets the same MRI or X-ray differently [30]. Recent data presented to the US Food and Drug Administration highlighted the issue of limited reproducibility in evaluating the SIJs in pelvic radiographs of patients suspected of having SpA. In the ABILITY-1 trial, patients with nr-axSpA were initially diagnosed by local radiologists or rheumatologists based on pelvic radiographs. However, a subsequent central reading for a different purpose revealed that 37% of these patients were reclassified as meeting the modified New York (mNY) criteria [31, 32]. Similarly, in the RAPID-axSpA trial, a comparable analysis resulted in the reclassification of 36% of the patients (26% meeting the mNY criteria and 10% classified as nr-axSpA) based on the central reading, in contrast to the local reading [33, 34].

The identification of sacroiliitis depends on radiographic imaging, which also determines the type as either nr-axSpA or r-axSpA/AS. This differentiation, which is solely based on structural alterations, remains pertinent in clinical settings, as certain treatments are currently only available to individuals with r-axSpA. Assessment of structural changes also gains importance since expert opinion on what constitutes bone marrow edema (BME) highly suggestive of axSpA has changed over time, given the growing evidence that false-positive BME in the SI joint is commonly seen in patients who do not have axSpA [35]. MRI lesion cut-offs that could indicate a structural lesion characteristic of axSpA have been analyzed to a lesser extent [36]. Studies examining the reliability and diagnostic performance of MRI and traditional radiography of the sacroiliac joints generally used separate scorings of radiographs and MR images and the individual scores were subsequently combined during analysis of these independent assessments [4, 6, 23, 37]. This does not accurately represent clinical settings in real life, since in everyday practice sacroiliac radiographs and MRI of patients are typically assessed by the clinician simultaneously to integrate all available information for diagnostic or classification reasons. In addition, radiologists combine all available images (a multimodal interpretation) to accurately interpret and report the radiological diagnosis. On this occasion, literature fails to address the question of whether patients can be better classified as radiographic or non-radiographic by simultaneously reading sacroiliac MR images and radiographs. Recently published two study used this model of simultaneous assessment of radiographs and magnetic resonance images, however they focused on diagnostic accuracy or diagnostic confidence and used a mixed study population of patients with axSpA and non-axSpA [29, 38]. Consequently, we designed this study to evaluate the notion that radiographs can be scored more reliably when evaluated alongside MR images as opposed to scoring them individually in a study population consisting of patients with axSpA diagnosis.

Therefore, the aim of this study was to evaluate the reliability of sacroiliac radiographic grading using the mNY criteria, both by assessing pelvic radiographs alone and by combining them with sacroiliac magnetic resonance imaging. We also analyzed changes in the classification of radiographic and non-radiographic axial spondyloarthritis, reclassifying it using two different reading methods.

## Patients and methods

### Patients

The analysis was made on the baseline data extracted from our single-center chronic low back pain cohort. The characteristics of our cohort have been comprehensively detailed in the previous publication [39]. In short, consecutive adult patients with chronic back pain lasting more than three months were recruited and screened for clinical, laboratory, and imaging findings suggestive of axial SpA. Patients meeting the ASAS classification criteria for axSpA were designated as the SpA group [40]. The same physicians performed anthropometric measurements and systematic clinical examinations for all eligible participants in the original study. Disease activity, and functional disability were assessed using the Turkish version of the Bath Ankylosing Spondylitis Disease Activity Index (BASDAI), and Bath Ankylosing Spondylitis Functional Index (BASFI). The physicians performed all these assessments without knowledge of imaging and laboratory data of the patients. MR images of the sacroiliac joints were obtained using a 1.5-T scanner (Siemens Aera; Siemens Medical Systems, Erlangen, Germany) with a specific imaging protocol. The image interpretation methods were described in detail in the original publication. This cohort consisted of patients meeting ASAS criteria for axSpA who were radiographic axSpA/AS and non-radiographic axSpA [20, 41].

The original study was conducted in compliance with the Good Clinical Practice Guidelines and was approved by the medical ethics committee. Informed consents have been obtained from all participants. Additional ethical approval was also obtained from our institution for this study and the ancillary analysis of the retrospectively extracted data from the original data (Istinye University, Human Research Ethics Committee, No:24–217).

## Imaging and scoring methods

### Only radiographs sessions

In this current analysis, the pelvic radiographs and sacroiliac MR scans were evaluated by two rheumatologists who did not participate in the previous cohort study. Two rheumatologists (KE and KO) independently graded the anonymized radiographs in three sequences: In the first sequence sets of radiographs (only Rx, without any knowledge of the MRIs) were graded in adherence with definitions of SIJ grades and radiographic lesion types outlined in the mNY criteria [42], which categorizes grade 0 as normal, grade 1 as suspicious changes, grade 2 as minimum abnormality (involving small localized areas with erosion or sclerosis, without affecting the joint width), grade 3 as unequivocal abnormality (involving moderate or advanced sacroiliitis with erosion, evidence of sclerosis, widening, narrowing, or partial ankylosis), and grade 4 as severe abnormality or total ankylosis. Radiographic sacroiliitis was defined as bilateral grade ≥ 2 or unilateral grade ≥ 3. The evaluations, focused only on radiographs (only Rx) were conducted on two separate occasions, 2 weeks apart, with the same radiographs presented in different random orders. During each reading session, readers were asked to evaluate their level of certainty using a numeric 0 to 10 scale.

### Consensus reading only radiographs

Following only Rx sessions, two rheumatologists and a senior radiologist (IY) scored the radiographs (based on mNY criteria) and recorded the results based on their collective consensus (at least two out of three readers agreed).

### Radiographs and magnetic resonance images combined sessions

After 4 weeks, the same assessment process was repeated, but this time the radiographs were scored (based on mNY criteria) in conjunction with simultaneous knowledge of anonymized sacroiliac MR scans (semi-oblique coronal and axial T1 weighted scans). This technique (multimodal image interpretation) involved a combination of MRI and radiographs, where the radiographs were meticulously graded alongside the insights provided by the MRI scans. During these sessions readers focused on structural changes (sclerosis, erosion, fat lesion, backfill and ankylosis) on MR scans and they were able to follow the projection of some of these changes on radiographs. Thus, we could assess how this data acquired from magnetic resonance images impacts radiographic scoring.

### Consensus reading combined radiographs and magnetic resonance imaging

Following radiography and magnetic resonance images combined sessions, two rheumatologists and a senior radiologist (IY) scored the radiographs (based on mNY criteria) in conjunction with simultaneous knowledge of anonymized sacroiliac MR scans (semi-oblique coronal and axial T1 weighted scans) and recorded the results based on their collective consensus (at least two out of three readers agreed).

All scores (2 “Rx only”, one “Rx consensus”, two “Rx + MRI” and one “RX + MRI consensus” sessions) were recorded in a standardized electronic data sheet.

### Statistics

To conduct an unbiased assessment, the images were anonymized and read in a random order in each session. The readers evaluating the images were not provided with any patient-specific information, such as demographic data or previous diagnoses. Nonetheless, all investigators were informed that the radiographs belonged to the SpA cohort population. The normality of the distribution of variables was evaluated using the Kolmogorov-Smirnov test. Mean and standard deviations were calculated.

An analysis of agreement involved cross-tabulation expressed by Cohen’s kappa or linear weighted kappa (κW) as appropriate. Intra-reader and inter-reader reliability for both reading techniques were measured using the weighted kappa statistic and agreement for specific classification (r-axSpA or nr-axSpA) was calculated.

The interpretation of all kappa values followed the criteria outlined by Landis and Koch. Specifically, a value of 0 indicated no agreement, 0–0.20 was considered to have slight agreement, 0.21–0.40 was deemed fair, 0.41–0.60 was classified as moderate, 0.61–0.80 represented substantial agreement, and 0.81–1 indicated nearly perfect agreement [43]. With a minimum accepted kappa of 0.6, a minimum expected kappa of 0.4, and a positive radiography ratio of 50%, the study population size was estimated to be over 130. This estimate was also consistent with the population size of other similar studies that have been published.

Statistical analyses were performed using SPSS Statistics version 22 (IBM, Armonk, NY, USA) and MedCalc v12.0 (Mariakerke, Belgium).

## Results

A total of 138 patients’ sets of pelvic radiographs and MR scans of the SI joints were available for the analysis. Demographic and clinical data of these patients were extracted and analyzed. Table 1 presents the demographic and clinical data of these patients.

**Table 1:** Demographic and clinical characteristics of patients with axial spondyloarthritis (*n* = 138)

Variable	Value
Age, mean (S.D.),	36.88 (8.77)
Symptom duration, mean (S.D.), years	9.27(6.58)
Male sex, n (%)	75 (54.3)
HLA-B27 positive, n (%)	88 (63.7)
Smoking, current/ever, n(%)	54 (39.1)
Family history for SpA, n (%)	32 (23.2)
Peripheral arthritis, n (%)	24 (17.4)
Uveitis, current/ever, n (%)	18(13.0)
Psoriasis, current/ever, n (%)	9 (6.5)
Inflammatory bowel disease, current/ever, n (%)	4 (2.8)
Treatment with cs/b/tsDMARDs, n (%)	59 (42.7)
BASDAI, mean (S.D.), 0–10	3.62 (2.28)
BASFI, mean (S.D.), 0–10	2.44 (2.29)
CRP, mg/dl, mean (S.D.)	14.38(20.92)

Abbreviations: Standard deviation, S.D: conventional synthetic, biologic, targeted synthetic disease modifying anti-rheumatic drugs, cs/b/tsDMARDs: BASDAI: Bath Ankylosing Spondylitis Disease Activity Index, BASFI: Bath Ankylosing Spondylitis Functional Index, C-reactive protein, CRP

Reader 1 demonstrated intra-rater reliability ranging from 0.40 to 0.52 for grading right and left radiographs according to the mNY criteria. Reader 2 also displayed similar, albeit slightly higher, kappa values, suggesting substantial intra-rater agreement (range 0.62–0.74) as shown in Table 2. The level of agreement among the readers, inter-reader reliability, was fair to moderate for both the first and second assessments of the radiographs, regardless of whether they were examined alone or in conjunction with MRI (Table 2). Also, the confidence levels of readers while scoring radiographs were relatively higher in MRI + radiograph combined technique compared to radiographs only technique. Mean confidence levels of Reader 1 were 7.69 ± 1.22 and 7.95 ± 1.07 for first and second reading of radiographs only, and 8.35 ± 0.81 and 7.90 ± 0.82 for MRI + radiograph combined technique, respectively. Whereas these values were 7.04 ± 1.77 and 8.10 ± 1.19 for radiographs only and 8.91 ± 0.78 and 8.94 ± 0.65 for MRI + radiograph combined technique for the Reader 2, respectively.

**Table 2:** Intra and inter-reader reliability of the reader 1 and reader 2

Intra-reader reliability	Reader 1 weighted kappa (CI 95%)	Reader 2 weighted kappa (CI 95%)
Right SI joint (Rx only)	0.52 (0.43–0.61)	0.62 (0.54–0.70)
Left SI joint (Rx only)	0.48 (0.39–0.57)	0.74 (0.67–0.81)
Right SI joint (Rx + MRI)	0.44 (0.33–0.54)	0.63 (0.53–0.73)
Left SI joint (Rx + MRI)	0.40 (0.29–0.52)	0.66 (0.56–0.75)
Inter-reader reliability	First reading weighted kappa (CI 95%)	Second reading weighted kappa (CI 95%)
Right SI joint (Rx only)	0.49 (0.39–0.59)	0.43 (0.33–0.53)
Left SI joint (Rx only)	0.47 (0.37–0.57)	0.42 (0.31–0.52)
Right SI joint (Rx + MRI)	0.30 (0.20–0.40)	0.47 (0.37–0.58)
Left SI joint (Rx + MRI)	0.40 (0.29–0.51)	0.46 (0.36–0.54)

Abbreviations: MRI: magnetic resonance imaging, Rx: radiographs, SI: sacroiliac

Cohen’s kappa values were also calculated for intra- and inter-reader reliability regarding classification of patients with r-axSpA vs. nr-axSpA. The MRI + radiograph combined technique revealed relatively higher Cohen’s kappa values for intra-reader reliability compared to radiographs only technique (Reader 1, 0.62 and 0.48 and Reader 2, 0.64 and 0.58, respectively). However, inter-reader reliability was fair for both combined and radiographs only techniques (Reader 1, 0.24 and 0.26, Reader 2, 0.39 and 0.33, respectively.)

**Fig. 1:**
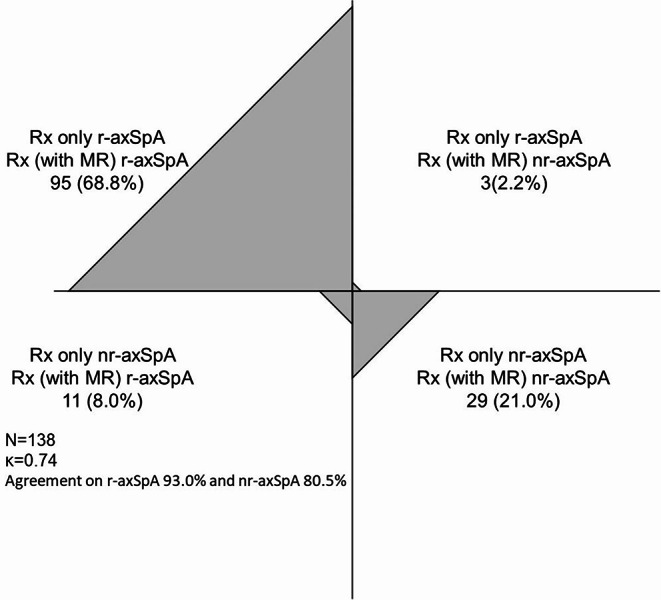
Graphic showing categorized patients as radiographic or non-radiographic axial spondyloarthritis according to the consensus sessions of radiograph only or the radiograph with MRI combined technique

Regarding the classification, both readers’ intra- and inter-reader agreements were moderate to substantial (Table 3). The kappa values and agreement were notably higher for the MRI + radiograph combined technique whereby radiographs are assessed in conjunction with the information obtained from MRI scans. Additionally, the agreement between the consensus readings using radiographs only (at least two out of three readers agreed) and the consensus readings using both radiographs and MRI scans was substantial with a kappa of 0.74, and agreement for the classification of r-axSpA was 93.0% and for nr-axSpA was 80.5%. In the consensus sessions, three patients classified as r-axSpA in the radiograph only reading were re-classified as nr-axSpA in the MRI + radiograph combined reading, while 11 patients originally categorized as nr-axSpA in the radiograph only reading were recategorized as r-axSpA in the MRI + radiograph combined reading (totally 10.2% recategorized) (Fig. 1).

**Table 3:** Agreement between individual readings and consensus readings regarding the classification r-axSpA vs. nr-axSpA

	Rx only consensus reading		
Inter-reader reliability	Consensus reading Kappa	Agreement positive r-axSpA	Agreement negative nr-axSpA
Reader 1 Rx only first reading	0.31	77.6	53.3
Reader 1 Rx only second reading	0.35	69.1	57.6
Reader 2 Rx only first reading	0.32	69.6	60.0
Reader 2 Rx only second reading	0.49	79.5	67.3
	Rx + MRI consensus reading		
	Consensus reading Kappa	Agreement positive r-axSpA	Agreement negative nr-axSpA
Reader 1 Rx + MRI first reading	0.44	87.2	57.1
Reader 1 Rx + MRI second reading	0.47	88.3	59.0
Reader 2 Rx + MRI first reading	0.55	90.9	64.3
Reader 2 Rx + MRI second reading	0.86	97.6	89.2

Abbreviations: MRI: magnetic resonance imaging, nr-axSpA: non-radiographic axial spondyloarthritis, r-axSpA: radiographic axial spondyloarthritis, Rx: radiographs

## Discussion

Our study showed that reliability of sacroiliac grading was fair to moderate regardless of using radiography alone or in combination with MR. Therefore, adding MR to the evaluation or multimodal interpretation did not substantially enhance the reliability of the readers. However, the confidence of the readers and agreement for the classification of radiographic and non-radiographic axSpA were marginally boosted. To the best of our knowledge such comparison of these reading techniques using our study design in patients diagnosed with axSpA was conducted for the first time. Previous studies investigating reliability were primarily focused on the accuracy of axSpA diagnosis, conducted in mixed patient populations with and without axSpA or analyzed separately acquired radiographic or MR scores which were subsequently combined during analysis. We think that the method we employed in this study closely reflects typical daily practice.

Conventional pelvic radiographs remain the primary method for classifying or diagnosing ankylosing spondylitis, as they are readily available, cost-effective, and have accumulated experience. Studies have shown that while alternative radiographic methods, like Ferguson angle and AP lumbar spinal radiographs, offer some theoretical benefits, they do not significantly outperform traditional AP pelvic radiographs [7, 44]. Therefore, traditional pelvic radiographs remain the standard for evaluating sacroiliitis severity according to the mNY criteria. However, their reliability is limited due to the complex anatomy of the pelvis and interference from surrounding soft tissues and bowel gas [7]. EULAR recommendations for the use of imaging in the clinical diagnosis and management of axSpA advise radiography of the SI joints as the first imaging technique if axSpA is suspected, despite these constraints and amidst debates advocating magnetic resonance imaging [45].

MRI of sacroiliac joints is reliable for the detection of structural changes like erosions even in patients who do not have acute inflammatory lesions in sacroiliac joints [22]. Although, AP pelvic radiography has a wide range of inter-observer agreement (κ: 0.19–0.79), in terms of presence of structural changes detected on sacroiliac joint MRI, the inter-observer agreement was better (κ: 0.331–0.468 for different structural changes) [4]. In our study, we sought the answer to the question of whether we can increase intra- and inter-observer agreement by reading radiographs with enhanced information gathered from MR scans compared to reading radiographs alone, but we found similar levels of reliability for grading sacroiliac joints according to mNY criteria. The main reason for this may be the low agreement levels of radiographs and MRI in detecting structural changes in sacroiliac joints, which was the result obtained in Poddubnyy et al.‘s study (κ: 0.109) [4]. Notwithstanding observers’ confidence levels were relatively higher in radiograph and MRI combined technique rather than radiographs only technique while grading sacroiliitis.

Two recently published reports also provided levels of confidence concerning the diagnosis of axSpA. These two studies exhibited some differences when compared to our study design (i.e. were carried out in groups with suspicion of axSpA -both with and without an axSpA diagnosis- and focused on diagnostic confidence and precision) [29, 38]. Ulas et al.’s study recorded a comparatively lower diagnostic confidence of readers in the evaluation of radiographs alone compared to the evaluation of both radiographs and MR (XR + MRI combined) concerning correct and incorrect diagnoses [38]. Similar findings were also reported by Pohlner et al. who demonstrated that a noticeably greater level of confidence was noted in evaluations combining radiographs and MRI compared to evaluations using only radiographs assessed with and without clinical information [29].

In Diekhoff et al. study, three expert radiologists graded the sacroiliac joints of 163 patients (89 with axSpA; 74 with degenerative conditions) according to the mNY criteria in 5 different conditions (Rx, CT, MRI, Rx + MRI, CT + MRI) [46]. Like ours, the inter-observer reliability level of the AP pelvic radiography assessment was moderate for structural lesions (κ:0.516). The study revealed that while assessing the sacroiliac joint for structural lesions, the consistency between different raters was notably greater when combining X-ray and MRI evaluations, with a reliability score of 0.668. In contrast, the reliability for X-ray evaluations alone was lower, with a score of 0.516. Furthermore, the reliability of MRI readings alone was comparable, scoring 0.695. The researchers concluded that when MR images are available, pelvic radiography provides minimal additional information [46]. Similarly, Ulas et al. underscored that the addition of radiographs to MRI did not improve the diagnostic confidence in the diagnosis of AxSpA in unexperienced and experienced readers [38]. In our trial, we determined that the reliability among readers, both within their own assessments and when comparing their evaluations to others, remained consistent whether they were examining X-rays alone or in combination with MRI scans, reflecting a moderate level of agreement. The initial aspect in which our study diverges from Diekhoff et al. is that we concentrated solely on structural alterations, without assessing inflammatory changes and diagnostic accuracy. Furthermore, whereas Diekhoff et al. concentrated on evaluating MR images of the sacroiliac joints in conjunction with X-ray evaluations, our method entailed assessing the radiographs based on the significant information obtained from MRI scans. This variation in methodology may account for why our results demonstrated moderate reliability in grading the sacroiliac joints, whether based on radiographs alone or when incorporating both radiographs and MRI collectively.

In another study by Protopopov et al. [37] radiographs and MR images were evaluated by two central readers for structural changes (based on the mNY criteria and based on an additional criteria with a lower score at least grade 2 unilaterally) by two independent readers. MR images were assessed by seven central readers for structural changes as well (who were different from radiographic readers). It was concluded that reliability of radiographic grading was moderate and MR assessment of sacroiliitis regarding the structural changes were more specific. Furthermore, the integration of the independently acquired data from radiographs and MR scans did not significantly enhance diagnostic performance. In our research, we achieved comparable reliability findings with the results of Protopopov et al. concerning radiographic grading, and the concurrent evaluation of radiographs and MR (multimodal interpretation model) did not improve the reliability of radiographic grading.

In a study conducted by Poddubnyy et al., researchers delved into the progression of radiographic sacroiliitis in patients diagnosed with early axSpA over a span of two years [13]. The findings revealed that the agreement level between two trained readers, tasked with grading sacroiliitis, was moderate (weighted κ values 0.51–0.59), echoing the results of our own research. Specifically, Poddubnyy et al. [13] reported a similar moderate level of agreement when it came to categorizing patients as either nr-axSpA or r-axSpA, with baseline agreement showing a kappa value of 0.59 and follow-up after two years reflecting a value of 0.67. In our study, we observed comparable moderate levels of intra-observer agreement while classifying patients as nr-axSpA or r-axSpA, whether using radiographs alone or a combination of radiography and MRI. The kappa values, specifically 0.48 and 0.62 for Reader 1, and 0.58 and 0.64 for Reader 2, indicated consistent reliability in these assessments. However, when it came to inter-observer reliability, the results were less favorable-falling into the realm of fair agreement as we classified patients as nr-axSpA or r-axSpA based on radiographs only or combined techniques. The kappa values in this aspect ranged between 0.26 and 0.39, highlighting the challenges in achieving consensus among different observers.

In the research conducted by Christiansen et al., a notable observation arose regarding the inter-observer reliability [11]. Their findings revealed that the agreement among different observers in categorizing patients into either nr-axSpA or r-axSpA was fair to moderate, with kappa values ranging from 0.36 to 0.55. This was consistent with the results we obtained in our own study. When we consider both the previous studies and our recent findings, it becomes clear that the level of consensus among observers remains at a fair to moderate standard when it comes to distinguishing between radiographic and non-radiographic axial spondyloarthritis. Moreover, this level of agreement does not significantly improve, even when the radiographic assessments are supplemented with guidance from MRI.

In another research conducted by Poddubnyy et al. [4], two trained readers took on the task of evaluating the pelvic radiographs and sacroiliac joint MRIs of patients with axSpA, working independently of one another. Following the mNY criteria, they found that a significant 68.3% of the patients exhibited at least grade 2 sacroiliitis on radiographs, while 69.6% showed similar findings on the MRI. Interestingly, both radiographs and MRI identified sacroiliitis in 57.1% of the patients examined. The level of agreement between the two modalities in detecting definitive sacroiliitis was deemed moderate, with a kappa statistic of 0.447. Clinically, this reflected in the patient classifications, revealing that 60.7% of them were identified as having r-axSpA based on radiographic findings, whereas a slightly higher percentage of 63.4% received the same classification when assessed through MRI.

In the DESIR cohort study conducted by Bakker and colleagues, it was revealed that when structural lesions were assessed using both MRI and radiography, a notable proportion of patients with nr-axSpA were redefined as having r-axSpA [23]. The findings indicated that between 8% and 22% of the patients were reclassified, depending on whether reader one or reader two conducted the evaluation. Furthermore, the consensus assessments, which incorporated both radiographs and MRI scans, demonstrated a significant level of agreement, with a kappa statistic of 0.74 in our study. In terms of classification, the agreement reached 93.0% for r-axSpA and 80.5% for nr-axSpA in our analysis. Notably, 11 individuals who were initially classified under nr-axSpA based solely on radiography were later identified as r-axSpA when MRI was included in the evaluation, resulting in a total reclassification rate of 10.2%.

Our study has some limitations. Firstly, only the level of radiographic sacroiliitis was assessed; additional details regarding structural alterations such as erosion, sclerosis, etc. would be beneficial. Nonetheless, given the significant inter-reader variability even in the fundamental grading of sacroiliitis, a more comprehensive assessment including various structural modifications appeared to be doubtful. Second, in terms of multimodal interpretation, a more precise technique for detecting structural changes, such as computerized tomography, would hold greater value. Nonetheless, the assessment of radiographs and MR of the sacroiliac joints might better represent everyday clinical practice. Third, clinical information might have enhanced the certainty of the diagnosis; however, the objective was to achieve an unbiased evaluation of the imaging findings.

In conclusion, the agreement level of radiographic scoring of sacroiliac joints was moderate. Adding simultaneous knowledge of sacroiliac MRI T1W scans to radiography does not substantially improve the intra- and inter-observer reliability levels but provides more confidence. Despite the use of MRI guidance, relying on the evaluation of sacroiliac joint radiographs or categorizing axSpA as either non-radiographic or radiographic based on anteroposterior pelvic radiography proves to be insufficiently reliable. These findings add to the ongoing discussion about whether radiography should serve as the initial imaging modality for suspected spondyloarthritis, or if it should be substituted with MRI or computed tomography. This also raises the question of whether individuals with axSpA should continue to be categorized as having non-radiographic or radiographic disease, based on a definition that is exclusively restricted to sacroiliac radiographs, or if they should be divided into subgroups with a more precise definition, such as axSpA with or without structural changes in the sacroiliac joints, based on advanced imaging techniques like CT and MRI.

## Data Availability

The data associated with this study are not publicly available but can be obtained from the corresponding author upon reasonable request.
